# Controlled, double-blind, randomized clinical trial to evaluate the impact of fruit juice consumption on the evolution of infants with acute diarrhea

**DOI:** 10.1186/1475-2891-4-23

**Published:** 2005-08-09

**Authors:** Sandra Valois, Hugo Costa-Ribeiro, Ângela Mattos, Tereza Cristina Ribeiro, Carlos Maurício Mendes, Fima Lifshitz

**Affiliations:** 1 Department of Pediatrics, Hospital Professor Edgar Santos, Universidade Federal Da Bahia, Salvador, Bahia, Brazil; 2 Department of Pediatrics, Sansum Medical Research Institute, Santa Barbara CA, USA

## Abstract

In order to assess the effects of juice feedings during acute diarrhea a double-blind, randomized study was performed in 90 children, mean age of 10 ± 4.28 months. Thirty patients with acute diarrhea were fed twice-daily 15 ml/kg of Apple Juice (AJ), 30 received White Grape Juice (WGJ), and 30 were given colored and flavored water (WA) as part of their age appropriate dietary intake. The duration and severity of diarrhea were the main endpoint variables of the study performed in a metabolic unit. The patients were similar among the 3 groups, had diarrhea for 50–64 hours prior to admission, and were dehydrated when admitted to the unit for study. Half of the patients in each group were well nourished and the others had mild to moderate degrees of malnutrition. Rotavirus infection was the agent causing the illness in 63% of the patients. The infants fed juice ingested 14–17% more calories than those given WA, (those receiving AJ and WGJ ingested 95 and 98 Calories/Kg/d respectively) whereas those receiving WA consumed 81 cal/kg/d). The increased energy intake was not at the expense of other foods or milk formula. The mean body weight gain was greater among patients receiving WGJ (+ 50.7 gm) as compared with the patients in the AJ group (+ 18.3 gm) or the patients fed WA (- 0.7 gm) (p = 0.08). The duration of the illness was longer in the infants fed juice as compared with those given WA (p = 0.006), the mean +/- SD duration in hours was 49.4 ± 32.6, 47.5 ± 38.9 and 26.5 ± 27.4 in patients fed AJ, WGJ and WA respectively. All patients improved while ingesting juice and none of them developed persistent diarrhea; most recovered within 50 hours of the beginning of treatment and less than one fourth had diarrhea longer than 96 hours in the unit. The fecal losses were also increased among the juice fed patients (p = 0.001); the mean ± SD fecal excretion in g/kg/h was 3.94 ± 2.35, 3.59 ± 2.35, and 2.19 ± 1.63 in AJ, WGJ and WA respectively. The stool output was highest during the first day of treatment among all the patients, though those fed AJ had the highest volume of fecal losses and those who received WA had the lowest stool excretion. After the first day of treatment the differences in fecal excretion were not significant. The ability to tolerate carbohydrates during the illness and immediately after recovery was similar among the 3 groups of patients. Intake of juices with different fructose/glucose ratios and osmolarities resulted in more fecal losses and more prolonged diarrhea as compared with water feedings, but the patients given juice ingested more calories and gained more weight, particularly among those being fed the juice with equimolar concentrations of fructose and glucose.

## Introduction

Worldwide, acute diarrhea represents one of the leading causes of death in children less than 5 years of age. The average duration of an acute episode of this illness is 5 days, but in some cases the diarrhea may persist more than 14 days. This leads to deterioration of the nutritional status and of the prognosis of the disease. Whereas 0.7% of acute diarrhea cases may be fatal, mortality may be 10 to 35% in the persistent diarrhea patients [1]. Previous studies have demonstrated that proper management of hydration and appropriate dietary intake during the illness can decrease the stool output, reduce the risk of prolonged diarrhea, and improve the nutritional status of patients [1-3].

The World Health Organization (WHO) has recommended home fluids, including fruit juices, as appropriate feedings and as a measure to improve fluid balance during acute diarrheal episodes in children [1]. On the other hand the Provisional Committee on Quality Improvement, Sub-committee on Acute Gastroenteritis and the Committee On Nutrition of the American Academy of Pediatrics recommended that the management of diarrheal disease in young children should include early feedings of age appropriate foods, while avoiding foods high in fat and feedings with simple sugars, including teas, juices and soft drinks [4,5]. ESPGHAN's working group on acute diarrhea endorsed similar recommendations. They recommended the use of a normal diet without restrictions, including lactose [3].

However, the recommendations for or against juice feedings during acute diarrhea have not been prospectively assessed. Previous data showed that not all fruit juices are equally absorbed. Some juices, like pear (PJ) and apple juice (AJ), which contain more fructose than glucose and sorbitol, are poorly absorbed as compared with white grape juice (WGJ) which contains equimolar concentrations of fructose and glucose, without sorbitol. We recently demonstrated that children challenged with a single serving of PJ or AJ during the recovery phase of diarrhea presented recurrence of loose stools, whereas those who received WGJ did not [6]. In other reports it has been shown that apple juice ingestion may be associated with chronic diarrhea [7].

In this double blind randomized clinical trial we measured the effects of juice intake during acute diarrheal illness. AJ, WGJ, and water feedings were given twice daily as part of an age appropriate dietary intake. While feedings of juice increased the stool loses and the duration of diarrhea, juice intake also contributed to a higher energy intake and increased weight gain, particularly among those fed the juice containing equimolar quantities of fructose and glucose.

## Patients and Methods

The study was conducted in a double-blind design. There were 90 infants with severe diarrhea admitted to the Fima Lifshitz Metabolic Unit at The University Hospital Professor Edgar Santos, Federal University of Bahia, Salvador, Bahia, Brazil. Thirty patients in each group were randomly assigned to receive one of three juice feedings: apple juice (AJ), white grape juice (WGJ) or water (WA). The composition of each one of them is shown in table 1. These were packaged by the manufacturer (Welch's in Concord MA) in identical bottles and were of the same appearance and color. The WA was colored and flavored to resemble juice. Thirty identically labeled bottles containing 300 ml each were provided per patient. A new bottle was opened for each of the twice daily feedings. The investigators involved with the care of the patients in the study were not aware of the code identifying the bottle content or of the type of juice that the infant was randomized to be fed. To establish the randomization list, permuted blocks of variable length, with four blocks for each group, were used in order to avoid imbalance between treatment groups.

**Table 1:** Carbohydrate Content of Fruit Juices

Juice	Osmolality mOsm/L	Fructose gm/dl	Glucose gm/dl	Sucrose gm/dl	Sorbitol gm/dl	Energy cal/dl
Apple	700	6.2	2.7	1.2	0.5	40.4
White Grape	1040	7.5	7.1	0.0	0.0	58.4
Water^x^	46	--	--	--	--	--

Modified from:

Hyams JS, Etienne NL, Leichtner AM, Theuer RC, Carbohydrate malabsorption following fruit juice ingestion in young children. Pediatrics 1988;82:64-8. Hardinge MG, Swarner JS, Crooks H. Carbohydrate in foods. J AM Diet Assoc 1965;46:197-204

^x^Colored/flavored to resemble juice

The patients fulfilled the following inclusion criteria: male, age 4 to 18 months, had an episode of acute diarrhea (defined as more than 3 watery stools in the previous 24 hours and of no more than 3 days duration prior to admission) and were moderately dehydrated. Patients presenting severe dehydration or other conditions or concurrent serious illness, and history of chronic diarrhea as well as those exclusively breast fed prior to the time of the illness were excluded from the study. An informed consent was elicited from the mothers of all patients admitted into the protocol. The study was approved by the Institutional review Board of the Hospital and of the University.

The patients were hydrated according WHO guidelines. They were treated with ORS given at a dose of 100 ml/kg over 6 hr. The maintenance hydration phase started once the acute dehydration was treated. This was continued throughout the duration of the illness. During this phase the patients received ORS solution on a volume to weight replacement of ongoing stool loses and vomit. After rehydration was achieved, the infants were started on their usual diet that consisted of age appropriate milk formula and feedings and complementary foods [8]. Additionally, all infants received a serving of 15 ml/kg of AJ, WGJ, or WA, twice daily (10 AM and 3 PM) throughout the diarrheal episode. Plain water was offered "ad libitum" between meals. Lactose free formula was utilized in two patients who had severe stool losses >10 ml/kg/hr during their milk formula feedings.

A Breath Hydrogen Test (BH2) was performed twenty-four hours after the illness improved, defined as two formed stools passed during 24 hrs., or no stools for 12 hrs. The patients were fasted for 6 hours and a juice feeding was given. Breath Hydrogen levels were measured every 30 minutes for 3 hours using a SC Microanalyzer (Quintron Instruments Co). A peak rise in H2 of at least 20 ppm was considered as a positive response [9].

Body weight was measured on admission, after rehydration, and daily thereafter until discharge. Nutritional assessment was determined using weigh-for-length with National Center for Health Statistics (NCHS) data as reference. Nutritional intake and the amount of fluids ingested were measured throughout the study. Stool weight, Urine volume, and Vomitus weight were quantitated using metabolic techniques and specially designed beds to accurately collect stool loses throughout the study. Breast milk intake was estimated by weighing the patients before and after breastfeeding. All stools were tested for pH and sugars by Clinitest tablets. Standard laboratory techniques were used for measurement of serum sodium, potassium and hemoglobin and hematocrit on admission, at 24 hours after the initiation of the study and as clinically indicated afterwards. Stool cultures for pathogens and rotavirus by ELISA were performed on admission. All patients were requested to return after one week for outpatient clinic follow-up. At this visit clinical evolution of each patient was recorded.

The following endpoint variables were quantified: duration of the illness, severity of diarrhea (assessed by the number, type and consistency of the stools) and the amounts of fecal loses measured as g/kg/day. Vomitus losses were also quantitated. The amount of fluid intake required to maintain fluid balance was also determined. Body weight changes were measured utilizing the weight of the patient attained after rehydration as compared with the one prior to discharge. The presence of carbohydrate intolerance was determined by the fecal pH and sugar excretion as well as by the breath hydrogen levels after juice intake.

The sample size was calculated by the Power program to ensure statistically significant differences on the stool output and duration of diarrhea [10]. The estimated sample size was 26 patients per group assuming a 30% clinical improvement, on the above outcomes, at a power of 80% at 0.05 significance. Data were analyzed by Analysis of Variance (ANOVA), when data distribution was not normal, a non-parametric test of Kruskal-Wallis were performed. Survival curves Kaplan-Meyers were used when appropriate [11].

## Results

The clinical characteristics and the laboratory data of the patients on admission are shown in tables 2 and 3. The patients in each of the 3 groups were similar in age, duration and severity of diarrhea, presence of fever and vomiting. Also there were no differences in the proportion of patients who received breast feedings or in their nutritional status. Over 46% of patients studied in each group, were well nourished, more than 33% showed mild body weight deficits (<1 SD) and the others had mild to moderate malnutrition (>2 SD). All patients presented some dehydration (mild to moderate), and required no intravenous hydration therapy. Differences in clinical characteristics were not significant among groups.

**Table 2:** Clinical Characteristics of Patients on Admission

	Apple Juice n = 30		White Grape Juice n = 30		Water* n= 30	
	
	χ	*SD*	χ	*SD*	χ	SD
Age (months)	10.27	4.75	10.27	4.14	11.09	4.00
Diarrhea Duration (hr)	56.37	33.90	64.17	38.95	53.47	33.35
Fever Duration (hr)	37.77	33.46	47.11	49.62	41.04	38.11
Vomiting (h)	41.57	35.45	46.97	39.54	44.33	31.44
Breastfeeding	13 (n)	43.3%	16 (n)	53.3%	09 (n)	30.0 %
Well Nourished	16 (n)	53.3%	15 (n)	50.0%	14 (n)	46.6%
Nutritional Risk**	08(n)	26.6%	10(n)	33.3%	15(n)	50.0%
Mild Malnutrition	03 (n)	10.0%	05(n)	16.6%	01(n)	3.4%
Moderate/Severe Malnutrition	03(n)	10.0%	00(n)	0.0%	00(n)	0.0%

There were no statistically significant differences among groups, Analysis of Variance (ANOVA) p > 0.05. Fever was considered above 37.5 C temperature. Nutritional risk indicated body weight deficit < 1 SD, Mild malnutrition < 2 SD and Moderate/Severe Malnutrition > 2 SD.

n = number of patients and % of patients in each category.

*Colored/flavored to resemble juice

**Table 3:** Laboratory Data of Patients on Admission*

	Apple Juice n = 30		White Grape Juice n = 30		Water^*^ n = 30	
	
	χ	*SD*	χ	*SD*	χ	*SD*
Serum Sodium (mEq/l)	142.03	5.45	140.96	3.51	140.51	5.39
Serum Potassium (mEq/l)	3.99	0.59	3.95	0.81	4.27	0.78
Hematocrit (%)	31.17	3.29	30.93	2.50	32.03	3.55
Hemoglobin (g/dl)	10.28	1.13	10.24	0.87	10.58	1.19
No Anemia	06(n)	20.0 %	02(n)	6.7 %	07(n)	23.3 %
Mild Anemia †	20(n)	66.7 %	26(n)	86.6%	20(n)	66.7 %
Severe Anemia†	04(n)	13.3 %	02(n)	6.7 %	03(n)	10.0 %

Rotavirus	19(n)	63.3 %	18(n)	60.0 %	18(n)	60.0 %
Parasites‡	05(n)	16.7%.	04(n)	13.3 %	01(n)	3.3 %

*There were no significant differences among groups by ANOVA p > 0.05.

† The criteria for mild Anemia was a hemoglobin less than 11.0 g/dl and for severe anemia was a hemoglobin less than 9 g/dl (WHO, 1989)

‡ The parasites detected were: Ascaris lumbricoides (4), Giardia lamblia (1) Blastocytes hominis (1) Entamoeba coli (1) and Cryptosporidium parvum (3).

n = number of patients and % of patients in each category

^x^Coloured/flavoured to resemble juice

The serum electrolyte levels on admission to the hospital were similar among the 3 groups of patients (Table 3). The hemoglobin (Hb) and hematocrit levels were also similar among groups, but two thirds of the patients exhibited mild degrees of anemia (Hb < 11 g), and in 9 infants there was a more severe degree (Hb < 9 g). In all instances iron supplementation was prescribed at the completion of the study. Rotavirus was identified in the stools of 55 of the patients, in 4 there was a pathogenic Escherichia Coli, in 10 there were parasites detected, and in the remaining 21 infants there were no stool pathogens identified.

The daily intake of the patients while in the study is shown in table 4. The amount of water, milk formula, and breast milk feedings did not differ among the groups. However, the infants given WGJ readily consumed more juice than those fed AJ or WA. The total energy intake was higher in the juice fed groups as compared with the WA one. The WGJ patients ingested an average of 17% more calories on a daily basis, and the AJ infants consumed a mean of 14% more than the WA group. The increased energy intake was not at the expense of the other foods; both milk formula and complementary foods were ingested in similar quantities among the 3 groups of patients. The mean body weight gain was also higher among the juice fed patients; there was a mean weight gain of 18.3 gm in the AJ fed patients and of 50.6 gm in those given WGJ, whereas there was a mean loss of body weight (- 7.0 gm) among the WA group patients (p = 0.08).

**Table 4:** Daily intake of patients throughout the study (Kcal/Kg/day)

	Apple Juice n = 30		White Grape Juice n = 30		Water^*^ n = 30		P value
		
	χ	*SD*	χ	*SD*	χ	*SD*	
Total Calories	95.84	22.42	98.65	30.52	81.43	23.09	0.02*
Milk Formula	54.49	23.43	50.21	34.57	52.68	17.93	0.81
Breast Milk	08.71	11.57	15.80	18.56	05.45	10.96	0.06
Water	30.67	9.25	30.27	09.57	26.98	9.37	0.25
ORS	45.52	31.17	39.12	25.10	25.10	17.91	0.01**
"Juices"	18.61	3.93	20.98	5.35	17.32^x^	4.37	0.01***
Total Liquids	157.90	38.26	158.42	50.35	127.70	28.25	0.001****
Complementary Foods	23.90	11.61	25.12	13.97	26.92	11.59	0.64

* Significant differences for Juice Groups vs Water, by ANOVA (Bonferroni)

** Significant differences for Water vs both juice groups

*** Significant differences for WGJ vs each other group

**** Significant Differences for Water vs each other group

^x ^Colored flavored water to resemble juice

Data are means +/- SD

**Figure 1:**
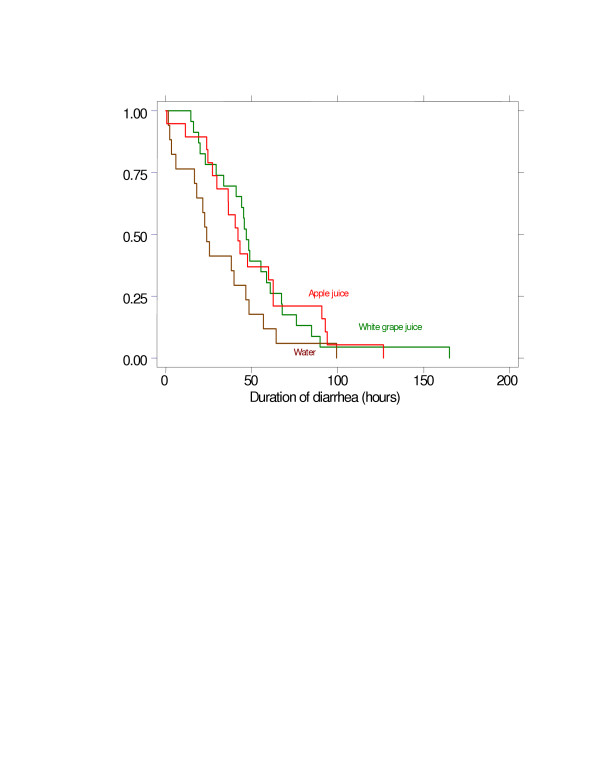
Survival analysis of total of diarrhea – Kaplan-Meier, per group. * Statistic difference were found, p < 0,05, among water group and apple juice (P = 0,03) or white grape juice (P = 0,00).

The duration of diarrhea differed among the 3 groups of patients (Table 5). The total duration of diarrhea from the start of the illness through their recovery was decreased among the WA fed patients as compared with the AJ and WGJ: groups 111.7 ± 48.2, 105.4 ± 44.9 and 80.0 ± 39.6 hours in AJ, WGJ and WA respectively. The differences in the duration of diarrhea were more marked while being treated in the hospital, the illness being shorter among patients given water instead of juice. However the majority of the patients recovered promptly regardless of the treatment given (Figure 1). Most of the patients improved within 50 hours after treatment was instituted, less than one forth of them had diarrhea persisting more than 96 hours and no one had it for more than 7 days.

**Table 5:** Duration of diarrhea in hours after randomization. Duration of diarrhea

	Mean	SD
Apple Juice	49.4	32.6
White Grape Juice	47.5	38.9
Water *	26.5	27.4

*Significant differences detect by Kruskal-Wallis p < 0.05. Water vs Juice groups. Data are hours +/- SD

Water coloured and flavored to resemble juice.

The severity of diarrhea also differed among the treatment groups (Table 6). The stool output among the WA group of patients was significantly decreased as compared with those fed juice. During the first day of treatment those fed water had a mean stool output of 40% less than those fed juice. With improvement the differences among the treatments were minimized, and were no longer significant after the 2^nd ^day of the illness. There were also significant differences in the severity of diarrhea among AJ and WGJ feedings (Table 6). During the first 24 hours of treatment of the illness the patients receiving AJ had more marked stool losses than those fed WGJ. The mean excretion of stools was 21% higher in AJ fed patients than in the WGJ fed group.

**Table 6:** Fecal losses throughout the study and on the first day after randomization

	**Total Losses (g/kg/hr)**		**First Day Losses g/kg/hr**	
	Mean	SD	Mean	SD

Apple Juice	3.94	2.35	4.13**	2.90
White Grape Juice	3.59	2.35	3.28**	2.39
Water^x^	2.19*	1.63	1.78***	1.80

*Differences Among groups Water vs each of the juice groups (Kruskal-Wallis test) (p = 0.001)

** Differences between WGJ and AJ (p = 0.02)

*** Differences between Water and each of the juice groups (p = 0.001)

^x^Coloured/flavoured to resemble juice

The stool excretion data were treated for possible confounding and co-variables which could play a role in determining the final results. The covariance analysis and the robust regression showed a possible influence of ORS intake in the differences detected among the 3 groups of patients. The stool output was not different when the ORS intake was adjusted for the 3 groups of patients and the urinary outputs were also similar. Vomitus losses were not different among the 3 groups of patients, vomiting being present primarily during the first 24 hrs. of treatment.

There were 2 patients who had severe diarrhea during the treatment period (>10 ml/kg/hr). These patients were in the AJ group, one of these patients had acid stools and none had carbohydrates in feces. These 2 patients were treated with a lactose free formula, with improvement in stool output. There were three other patients who showed carbohydrate intolerance with acid stools or sugars in feces among the 3 groups, they improved without any dietary modifications.

Vomiting was present in all groups of patients: 22 (AJ), 26 (WGJ), and 19 (WA) during the first day of treatment; nevertheless this did not represented a limitation for Oral Rehydration Therapy (ORT) or feeding regimen and no patient was withdrawn or shifted to another therapy due to vomiting.

The response to juice feedings, as determined by breath hydrogen excretion after improvement of the illness, was not different among the 3 groups of patients. Most infants did not show breath hydrogen excretion levels above 20 ppm; and there were 17, 16 and 10 patients among the AJ, WGJ and WA groups respectively, who failed to show BH2 levels above 5 ppm at any time. Only 18 patients demonstrated a delta BH2 level increase above the basal value of more than 20 ppm. Eight of them were given AJ, 6 were fed WGJ and 4 received WA. There were 11, 16, and 13 patients in each group who exhibited a BH2 value above 20 ppm independent of the delta differences from basal among the WGJ, AJ and WA fed groups respectively. The differences in BH2 levels among groups were not significant.

## Discussion

This is the first double blind prospective study designed to evaluate the effects of fruit juice feedings during diarrheal disease in young children. Two commonly available juices were selected and compared with water intake, as part of an age appropriate dietary intake. One juice contained equimolar quantities of glucose and fructose (WGJ) and the other one provided a higher fructose to glucose ratio and contained sorbitol (AJ). All patients improved while being fed water or any of these 2 juices. However those receiving juice had more stool losses than those fed water, highest being among the patients fed AJ. This was a significant finding during the first day of treatment, not thereafter. On the other hand the children fed juice ingested more calories and gained more weight than those fed water, those fed WGJ having the best response.

Acute gastroenteritis continues to be a common illness among infants and children worldwide. The disease causes an estimated 2 million deaths annually among children in the developing world. In the United States diarrhea accounts for more than 1.5 million outpatient visits, 200,000 hospitalizations and 300 deaths per year [12]. Children younger than 5 years of age are at much higher risk of death from diarrhea than older children and adults [13]. Infants younger than one year of age are particularly susceptible to this disease and are at the highest risk of death, 43% to 78% of mortalities from the illness among children less than 5 years of age occur in infants less than one year [13-15].

Although the number of children currently dying from diarrhea continues to be unacceptably high, it is substantially lower than the 5 million deaths estimated 20 years ago [16]. The critical factors accounting for the reduction in mortality rates from this illness include widespread use of oral hydration solutions and the proper nutritional rehabilitation of sick infants [17].

The American Academy of Pediatrics has emphasized the importance of oral hydration and early nutritional support to aid these patients safely and effectively through the diarrheal episode [5]. A rapid realimentation with age appropriated foods and an unrestricted diet is recommended, as soon as dehydration is corrected. Nursing should be continued for those infants being breast fed and a standard full strength formula to be given to those formula fed children. The old concept of "Bowel Rest" has no scientific validity and it can serve to aggravate and increase the risks of the disease [18]. Apart from the undesirable metabolic effects of even brief fasts, withholding oral intake may further compound the intestinal absorptive processes and may lead to deterioration of the nutritional status of the patient [19]. Even though feedings increase the stool output and diarrhea, children who are fed attain higher body weights at the end of the illness than children who are not fed. This was evident in this study. Patients who were fed juice as part of the nutritional intake during the diarrheal illness had a higher body weight at recovery than those fed water, although they exhibited larger stool losses during the first 24 hours of nutritional rehabilitation.

Diarrhea, like other infections, decreases the appetite and sick infants often reject most foods, although breast milk is better accepted [20]. The lack of appetite may be mediated by interleukin 1, a hormone released by the white cells after infection [21]. The intensity of anorexia may not necessarily correlate with the severity of the illness. A child may lose his or her appetite with even mild diarrhea, with anorexia lasting from a few hours to several days [22]. As much as 20% to 70% of food available may be wasted or not eaten, during bouts of diarrhea [22]. Thus feedings of a well accepted available energy source might be desirable and necessary to enhance the nutrient intake of sick infants and young children. The patients in this study readily consumed fruit juice, and the intake of this food did not displace the consumption of other nutrients. Juice feedings resulted in a higher energy balance, particularly among the infants fed WGJ. Infants fed juice ingested 14–17% more calories than those given WA, AJ and WGJ ingested 95 and 98 calories/kg/d respectively, whereas those receiving WA consumed 81 calories/kg/d.

Previous data showed that fruit juices differ in carbohydrate composition and that juices containing equimolar concentrations of glucose and fructose were best absorbed throughout the first 5 years of life [7,23]. Similarly we have previously shown that this type of juice is better tolerated after recovery from acute diarrhea [6]. The present study confirmed that this juice was better suited during the acute stages of the illness. The fecal losses associated with consumption of WGJ juice during the treatment of acute diarrhea were lower than those observed during feedings of juice containing higher fructose to glucose ratios and sorbitol. However, the stool output was highest only during the first day of treatment, with differences in stool output rapidly disappearing with recovery from the illness. All patients improved within 3–4 days while ingesting juice and none of them developed persistent diarrhea. The ability to tolerate carbohydrates was also similar among the 3 groups of patients.

The patients were given ad libitum up to 15 ml/kg/dose of juice twice daily throughout the study. This dose of juice exceeded the recommended allowance by the AAP-CON [4] which limits the intake of juice to one serving of 4–6 oz per day to children of this age. However by allowing the patients to ingest at will the high energy drink during the illness, they did not consume the full amount of juice offered, they only ingested approximately 17 to 21 ml/kg/day, those receiving WA consuming the lesser amounts. The patients given juice feedings also ingested more fluids. One can speculate that fluid intake was higher due to fecal losses replacement and/or thirst induced by juice. A covariance analysis and a robust regression to determine a possible confounding factor did not support that possibility. No differences were found among groups by adjusting the stool output to ORS or fluid intake. However, the ORS volume represented the most important fluid intake, indicating that diarrhea duration and stool losses were the consequence of this finding.

The maintenance of a positive energy balance during the illness may be of particular importance for the vulnerable infant who is at a higher nutritional risk even before developing diarrhea [19,22]. This illness is considered to be one of the most important risks for the development of malnutrition [24]. Diarrhea and other infections affect the body's economy through a number of mechanisms including the decreased absorption of nutrients [22,24,25]. The provision of simple carbohydrates in a balanced proportion, as present in some juices, may facilitate energy balance even during the illness and may be positive for the infant's nutritional rehabilitation [6]. The ingestion of juice during the acute episode of diarrhea provided a higher average energy intake than that of those fed WA (+ 12 cal/kg/day for AJ and 18 cal/kg/day for WGJ). However ingestion of larger amounts of fruit juice has been associated with prolongation of diarrhea [7]. Additionally ingestion of juices containing high fructose and sorbitol may also be associated with other negative consequences, i.e. colic [26] and increased energy requirements [27].

The transient malabsorption during the acute phase of the illness may be overcome by absorptive advantages of the carbohydrate composition of specific feedings [6]. Similar results were found with amino-acid based ORS [28] and with the low-osmolality ORS [16,29,30], though there was a negative role of high osmolality solutions. However the most important therapy for the sick infant is the rapid rehydration, the maintenance of fluid and electrolyte balance and the provision of adequate feedings.

## Conclusion

Feedings of juices with different fructose/glucose ratio, with or without sorbitol and osmolality levels resulted in more fecal losses in the first 24 hours of diarrhea as compared with water feedings. However the patients given juice ingested more calories and gained more weight, particularly those being fed the juice with equimolar concentrations of fructose and glucose without sorbitol. All patients recovered with appropriate treatment without anyone developing persistent diarrhea. Our data strongly support the present recommendation of maintaining normal dietary habits during acute diarrheal episodes. However, juice choices may vary in its effect, as they vary in their composition; thus these differences should be considered when recommendations are made for feeding sick infants.
